# The Characteristics and Regulatory Mechanisms of Superoxide Generation from eNOS Reductase Domain

**DOI:** 10.1371/journal.pone.0140365

**Published:** 2015-10-14

**Authors:** Hu Peng, Yugang Zhuang, Yuanzhuo Chen, Alicia N. Rizzo, Weiguo Chen

**Affiliations:** 1 Department of Emergency Medicine, Shanghai Tenth People’s Hospital, Tongji University, Shanghai, China; 2 Division of Pulmonary, Critical Care, Sleep and Allergy, Department of Medicine, University of Illinois College of Medicine, Chicago, Illinois, United States of America; University of Kentucky, UNITED STATES

## Abstract

In addition to superoxide (O_2_^.-^) generation from nitric oxide synthase (NOS) oxygenase domain, a new O_2_^.-^ generation site has been identified in the reductase domain of inducible NOS (iNOS) and neuronal NOS (nNOS). Cysteine S-glutathionylation in eNOS reductase domain also induces O_2_^.-^ generation from eNOS reductase domain. However, the characteristics and regulatory mechanism of the O_2_^.-^ generation from NOS reductase domain remain unclear. We cloned and purified the wild type bovine eNOS (WT eNOS), a mutant of Serine 1179 replaced with aspartic acid eNOS (S1179D eNOS), which mimics the negative charge caused by phosphorylationand truncated eNOS reductase domain (eNOS RD). Both WT eNOS and S1179D eNOS generated significant amount of O_2_^.-^ in the absence of BH4 and L-arginine. The capacity of O_2_^.-^ generation from S1179D eNOS was significantly higher than that of WT eNOS (1.74:1). O_2_^.-^ generation from both WT eNOS and S1179D eNOS were not completely inhibited by 100nM tetrahydrobiopterin(BH4). This BH4 un-inhibited O_2_^.-^ generation from eNOS was blocked by 10mM flavoprotein inhibitor, diphenyleneiodonium (DPI). Purified eNOS reductase domain protein confirmed that this BH4 un-inhibited O_2_^.-^ generation originates at the FMN or FAD/NADPH binding site of eNOS reductase domain. DEPMPO-OOH adduct EPR signals and NADPH consumptions analyses showed that O_2_^.-^ generation from eNOS reductase domain was regulated by Serine 1179 phosphorylation and DPI, but not by L-arginine, BH4 or calmodulin (CaM). In addition to the heme center of eNOS oxygenase domain, we confirmed another O_2_^.-^ generation site in the eNOS reductase domain and characterized its regulatory properties.

## Introduction

Endothelial nitric oxide synthase (eNOS, type III) is an important enzyme that is involved in a variety of fundamental functions such as cardiovascular tension regulation, proliferation of vascular smooth muscle cells, leukocyte adhesion and platelet aggregation [1–2]. The eNOS gene undergoes co-translational *N*-myristoylation and post-translational cysteine palmitoylation in the nuclear unit and Golgi, respectively [3–4]. Endothelial NOS (eNOS, type III) contains two functionally independent domains: an N-terminal oxygenase domain and a C-terminal reductase domain. In the eNOS oxygenase domain, there are sites that bind BH4 and L-arginine and convert L-arginine into L-citrulline, generating nitric oxide (NO). In the eNOS reductase domain, there are FMN and FAD/NADPH binding sequence and they are structurally and functionally similar with NADPH-cytochrome P-450 reductase. Between the oxygenase and reductase domains, there is an amino acid sequence binds to calmodulin (CaM). This region is necessary for maintaining the eNOS dimer structure and normal function.

Early in vitro studies demonstrated that all three kinds of NOS produced superoxide in L-arginine and BH_4_-depleted condition [5–7]. Functional studies showed that superoxide (O_2_^.-^) formation from eNOS occurred primarily at the heme center of its oxygenase domain. This O_2_^.-^ synthesis from eNOS requires Ca^2+^/CaM and is primarily regulated by BH_4_ rather than L-arginine[8]. The eNOS reductase domain contains 684 amino acids and there are two major subdomains: a FAD/NADPH binding sequence (758–1004), which generates the electrons and delivers them to the heme center of oxygenase domain of another monomer; two FMN binding sequences (from 522 to 705), which connect with the CaM binding sequence[9]. Glutamine 894, a reductase domain amino acid, polymorphisms changed eNOS response to L-argnine and further affected eNOS activity[10]. Recent study showed that S-glutathionylation of cysteines in eNOS reductase domain reversibly decreased eNOS activity and increased O_2_^.-^ generation. However, whether these cysteines glutathionylation-mediated O_2_^.-^ generation has not yet been fully investigated[11]. Additionally, a iNOS reductase domain subcloning study demonstrated that the FAD/NADPH and FMN binding sequence was another superoxide generation site in iNOS [12]. As all three isoforms of NOSs have FMN and FAD/NADPH binding sequence in reductase domains, it would be interesting to determine the O_2_^.-^ generation location and regulatory properties in eNOS reductase domain.

ENOS activity is regulated by cofactors as well as several critical amino acid modifications [13]. For example, the phosphorylation target of Akt is bovine Serine 1179 (Serine 1177 of human eNOS). Serine 1179 phosphorylation results in enhancement of eNOS activity and increased sensitivity to Ca^2+^ and CaM. Mutation of S1179 to aspartic acid (S1179D), which mimics the negative charge caused by phosphorylation, also increases eNOS activity [14–15]. Additionally, cytochrome C reduction studies demonstrate enhanced electron flux through the reductase domain of S1179D bovine eNOS compared to wild type eNOS [16]. Early in vitro studies demonstrated that heat shock protein 90 (HSP90), and not Serine 1179 phosphorylation, modulates O_2_^.-^ generation in endothelial cells using DMPO spin trapping [17]. However, recent studies indicate that O_2_^.-^ production in EC is regulated by eNOS Serine 1179 phosphorylation through two independent pathways: a direct pathway and an indirect pathways [18–21].

In this study, we utilized a stable spin trap probe (DEPMPO) with EPR to investigate whether the eNOS reductase domain has the potential to generate superoxide. We also aimed to determine if the classic eNOS cofactors (BH4, L-arginine and Ca^2+^/CaM) and Serine 1179 phosphorylation affect this O_2_^.-^ generation capability. Our results indicate that there is a O_2_^.-^ generation center in the FMN and FAD/NADPH binding site of the eNOS reductase domain. The O_2_^.-^ generation from the eNOS reductase domain is not affected by BH4, L-arginine or Ca^2+^/CaM. However, it is regulated by Serine 1179 phosphorylation and by flavoprotein inhibition.

## Materials and Methods

### Materials

Calcium chloride (Ca^2+^) and Calmodulin (CaM), >95% pure, were purchased from Sigma Chemical (St. Louis, MO). 2',5'-ADP-Sepharose 4B was the product of Amersham Pharmacia Biotech (Piscataway, NJ). L-[^14^C]arginine was from NEN (Boston, MA). NADPH, tetrahydrobiopterin, *N* ^G^-nitro-L-arginine methyl ester (L-NAME), diphenyleneiodonium (DPI) and other reagents were purchased from Sigma unless otherwise indicated. Both bovine WT eNOS pcDNA3 and S1179D eNOS pcDNA3 plasmids were purchased from www.addgene.com.

### ENOS Purification

Both bovine WT eNOS and S1179D eNOS pcDNA 3 plasmid were used as templates and performed PCR. The forward primer was: 5’- CGGAATTCAACATGGGCAACTTGAAGAGTGTGGGCCAG-3’ and the backward primer is 5’-GCTCTAGATCATCAGGGGCCGGGGGTGTCTGG-3’. The bovine eNOS reductase domain (521–1205) PCR forward primer was: 5’-CGGAATTCAACAAAGCAACCATCCTGTACG-3’ and the backward primer is: 5’-GCTCTAGATCA TCAGGGGCCGGGGGTGTCTGG-3’ (start codon and stop codon was underlined). The PCR products were digested with EcoRI and XbaI and the product was subcloned into pCW plasmid [7, 22–23]. The resulting pCW plasmid was transformed into E.Coli (BL21) and cultured overnight. After harvesting the E.Coli by centrifugation, the bacteria pellet was homogenized in buffer A, which contains 50 mM Tris·HCl, pH 7.6, 0.1 mM EDTA, 150mM NaCl, 0.1mM DTT, 10% glycerol and protease inhibitor cocktail solution (Sigma). After being centrifuged (16,000 x *g*, 10 min) at 4°C, the supernatant was applied to a 2',5'-ADP-Sepharose 4B column pre-equilibrated in buffer A. The column was extensively washed with buffer A containing 600 mM NaCl and Tris·HCl buffer (50 mM, pH 7.6). The protein was then eluted with 5 mM AMP in 50 mM Tris·HCl (pH 7.6). The eluate was washed and concentrated using Centricon-100 (Amicon) concentrators. Further purification was performed using FPLC. The final purified WT and S1179D eNOS proteins were isolated from the fractions which had high absorption at 280nm and 408nm. Protein content of the preparations was assayed with the Bradford reagent (Bio-Rad) using bovine serum albumin as standard. The purity of eNOS was determined by SDS-PAGE and visualized with Coomassie brilliant blue (R-250, Bio-Rad) staining. The purified eNOS preparations exhibited one prominent band on gels with a molecular mass of 135 kDa. Purified eNOS samples were stored in 50 mM Tris·HCl (pH 7.6) buffer with 10% glycerol at -80°C.

### L-[14C]arginine to L-[14C]citrulline Conversion Assay

ENOS-catalyzation of L-[^14^C]arginine to L-[^14^C]citrulline conversion was monitored in a total volume of 200 μl of buffer containing 50 mM Tris-HCl, pH 7.6, 2 μM L-[^14^C]arginine, 0.5 mM NADPH, 0.5 mM Ca^2+^, 10 μg/ml calmodulin, 10 μM BH_4_, and 10μg/ml purified eNOS. After five minutes incubation at 37°C, the reaction was terminated by adding 3 ml of ice-cold stop buffer (20 mM Hepes, pH 5.5, 2 mM EDTA, 2 mM EGTA). L-[^14^C]Citrulline was separated by passing reaction mixtures through Dowex AG 50W-X8 (Na^+^ form, Bio-Rad) cation exchange columns and quantitated by liquid scintillation counting[24].

### EPR Spectroscopy and Spin Trapping

Spin-trapping measurements of oxygen free radicals were performed in 50 mM Tris-HCl buffer, pH 7.6, containing 0.5 mM NADPH, 0.5 mM Ca^2+^, 10 μg/ml calmodulin, 400nM purified eNOS, and 20 mM spin trap DEPMPO. EPR spectra were recorded in a disposable micropipette (50μl, VWR Scientific) at room temperature (23°C) with a Bruker EMX spectrometer operating at X-band with a high sensitive (HS) cavity (Bruker Instrument, Billerica, MA)using a modulation frequency of 100 kHz, modulation amplitude of 0.5 G, microwave power of 20mW, and microwave frequency of 9.863GHz as described [12, 25]. The central magnetic field was 3510.0 Gauss (G) and the sweep width was 140.0 G. The time constant was 163.84 ms, the sweep rate was 40.96 ms, and the receiver gain was 2×10^6^.

### NADPH Consumption by eNOS

NADPH oxidation was measured spectrophotometrically at 340 nm [26]. The reaction systems were the same as described in EPR measurements, and the experiments were run at room temperature. The rate of NADPH oxidation was calculated using a molar extinction coefficient of 6.22 /mM/cm

### Statistics

Data are expressed as means ± SE. Comparisons were made using a two-tailed paired or unpaired Student's *t*-test. Differences were considered to be statistically significant at *P* < 0.05.

## Results

### Expression of eNOS and Activity Analysis

**Fig 1:**
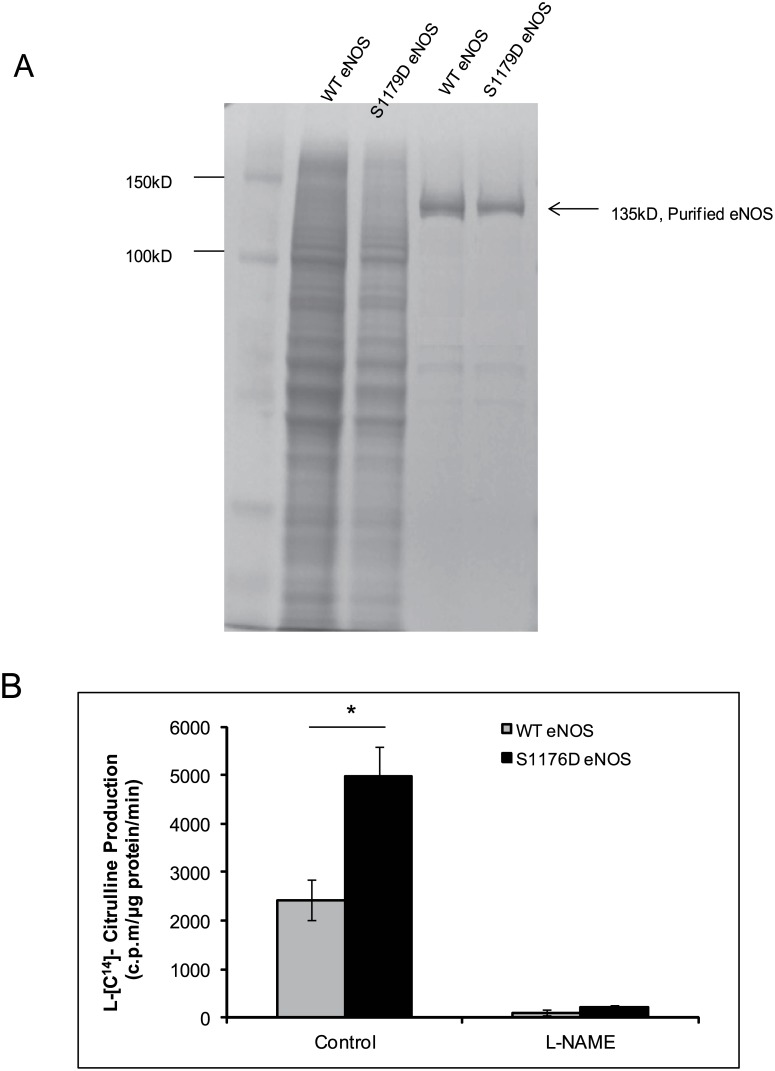
Mutation of Serine1179 of eNOS enhanced eNOS activity. (A) The bovine WTeNOS and bovine S1179D eNOS plasmid were expressed in E.Coli. The over-expressed eNOS proteins have been purified by FPLC as indicated in material and methods; the purity of protein was determine by coomassie bright blue stain after 1μg protein were run on SDS-PAGE gel. (B) eNOS activity was assayed by measuring the conversion of L-[^14^C]arginine to L-[^14^C]citrulline. The preparation showed typical eNOS characteristics with L-NAME-inhibitory activity (**, P< 0.01; *, P<0.05 compared with WT eNOS, n = 4).

Both wild type and S1179D eNOS were expressed and purified from E. Coli. In a culture of 2 liters, approximately 3.0–4.0 mg of eNOS was typically recovered using 2'5'-ADP Sepharose 4B chromatography. Further purification was performed using FPLC. The final purified products were identified by SDS-PAGE gel (Fig 1A). Previous studies demonstrated that eNOS has higher activity after serine 1179 phosphorylation or Serine 1179 mutation into aspartic acid (S1179D eNOS) [14, 27]. L-citrulline assay demonstrated that WT eNOS can convert L-[14C] arginine to L-[14C] citrulline at a rate of 2428.6±420.5 c.p.m /μg protein/min. S1179D eNOS can convert L-[14C] arginine to L-[14C] citrulline at a rate of 4968.7±626.6 c.p.m /μg protein/min. Both catalytic activities of WT eNOS and S1179D eNOS were blocked by the NOS inhibitor, L-NAME (1 mM) at 103.6±50.2 and 217.9±50.18 c.p.m/μg protein/min, respectively (Fig 1B).

### BH4 Specifically Inhibited O_2_^.-^ Generation from eNOS

**Fig 2:**
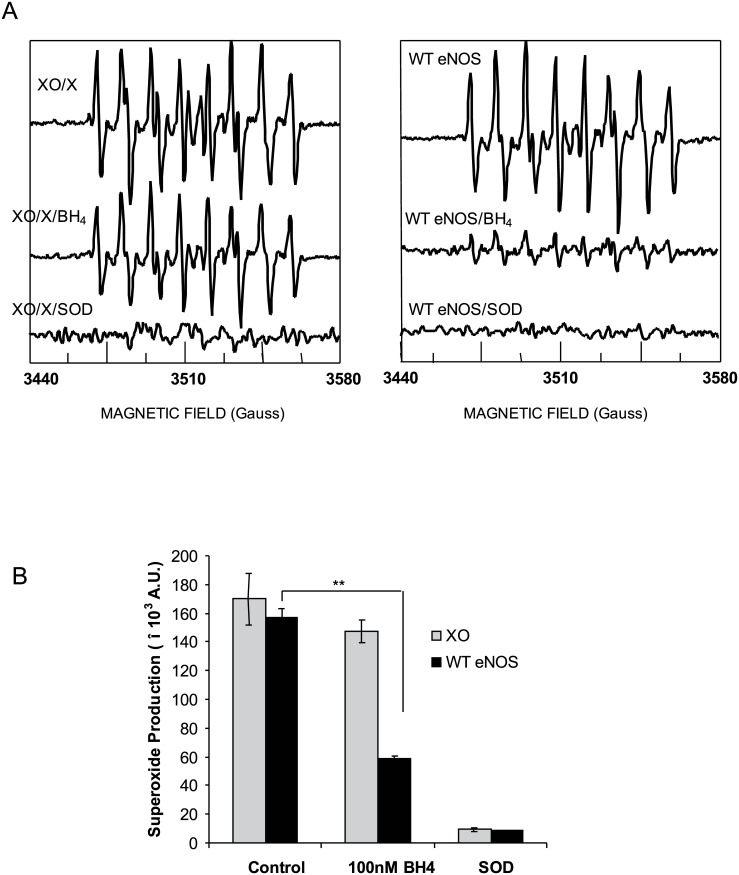
BH4 specifically inhibited O_2_^.-^ generation from eNOS. (A) A typical DEPMPO-OOH adduct signal was detected by a mixture of 0.005U/mL XO and 1mM xanthine, 20mM DEPMPO in 50mM Tris-HCl buffer, pH 7.6, This DEPMPO-OOH adduct signal was not inhibited by 100nM BH4. (B) 4μg eNOS protein mixed with 20 mM DEPMPO in 50 mM Tris-HCl buffer, pH 7.6, containing 0.5 mM NADPH, 0.5 mM Ca^2+^(calcium chloride), 10 μg/ml calmodulin (CaM). Similar DEPMPO-OOH adduct signal was recorded by EPR at 3440–3580 Hz range. Different with XO /Xanthine system, DEPMPO-OOH adduct signal generated from eNOS was significantly but not completely inhibited by 100nM BH4. Both of DEPMPO-OOH adduct signal generated from XO /Xanthine system and eNOS were completely inhibited by SOD (200 units/mL). The EPR spectra were representative spectra from five independent experiments and the density was presented as Mean ±SD.

Previous studies showed that all three isoforms of NOS, including eNOS generated O_2_^.-^[8]. To better determine O_2_^.-^ generation from eNOS, we first characterized our DEPMPO and xanthine oxidase/xanthine measurement system. DEPMPO which has a higher affinity for O_2_^.-^ than DMPO and its O_2_^.-^ adduct, DEPMPO-OOH is also more stable than the DMPO-O_2_^.-^ adduct[28]. Xanthine oxidase/xanthine (XO/X) is a well-known O_2_^.-^ generation system[29]. A characteristic DEPMPO-OOH adduct signal formed at the range of 3440–3580 Gauss after 20mM DEPMPO was added into a xanthine oxidase/xanthine (XO/X) mixture (Fig 2A left up-trace). To exclude the possibility of BH4 functioning as an O_2_^.-^ scavenger, 100nM BH4 did not affect the DEPMPO-OOH adduct signal generated from XO/X system (Fig 2A left middle-trace), which was consistent with previous observation[30]. Compared to XO/X system, 4μg eNOS generated similar density of DEPMPO-OOH adduct signal in the absence of L-arginine and BH4 (Fig 2A right top trace and Fig 2B). In contrast to DEPMPO-OOH adduct signaling generated from XO/X system, DEPMPO-OOH adduct signaling from eNOS was significantly inhibited by 100nM BH4 (Fig 2A right middle trace and Fig 2B). BH4 did not non-specifically scavenge the O_2_^.-^ until the concentration reached 500nM (data not shown). Moreover, DEPMPO-OOH adduct signals from both the XO/X system and from eNOS were significantly abolished by 200 units/mL SOD, indicating that these DEPMPO-OOH adduct signals were products of O_2_^.-^ (Fig 2A bottom traces and Fig 2B).

### BH4 Significantly but Not Completely Inhibited O_2_^.-^ Generation from eNOS

**Fig 3:**
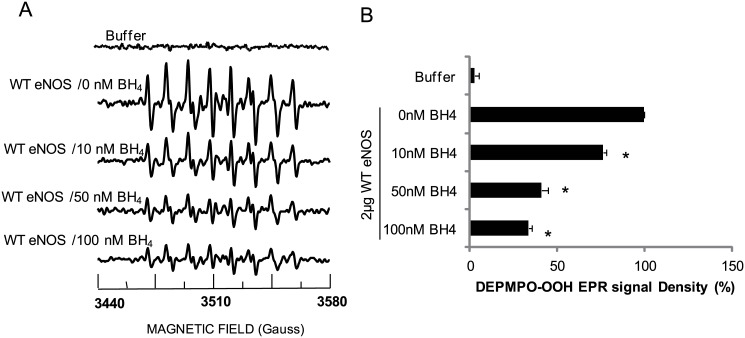
O_2_^.-^ generated from eNOS was significantly but not completely inhibited by BH4. 2μg eNOS protein mixed with 20 mM DEPMPO in 50 mM Tris-HCl buffer, pH 7.6, containing 0.5 mM NADPH, 0.5 mM Ca^2+^, 10 μg/ml CaM and different indicated concentration of BH4. (B)Statistical assay showed that DEPMPO-OOH adduct signals generated from eNOS were significantly inhibited by 10nM BH4 and decreased as dose-dependently till 100nM BH4 (n = 4).

BH4 is a critical regulator for O_2_^.-^ generation from eNOS, which binds with oxygenase domain. We next investigated the inhibitory effect of BH4 on O_2_^.-^ generation from eNOS[31]. A prominent DEPMPO-OOH adduct signal was recorded by EPR spectrum when 2μg WT eNOS was incubated with 20mM DEPMPO in the absence of L-arginine and BH4 (Fig 3A second trace). This O_2_^.-^ generation from WT eNOS was significantly inhibited by BH4 in a concentration dependent- manner (from 10nM to 100nM, Fig 3A third to fifth traces). However, BH4 did not completely inhibit O_2_^.-^ generation from WT eNOS even as BH4 concentration increased to 100nM or higher (Fig 3 fifth trace). Considering that BH4 binds with eNOS oxygenase domain, we hypothesized that eNOS may contain another O_2_^.-^ generation site located outside of the eNOS oxygenase domain. BH4 had similar inhibitory effect on O_2_^.-^ generation from S1179D eNOS (Data not shown).

### O_2_^.-^ Generated from the eNOS Reductase Domain Was Regulated by Serine 1179 Phosphorylation

**Fig 4:**
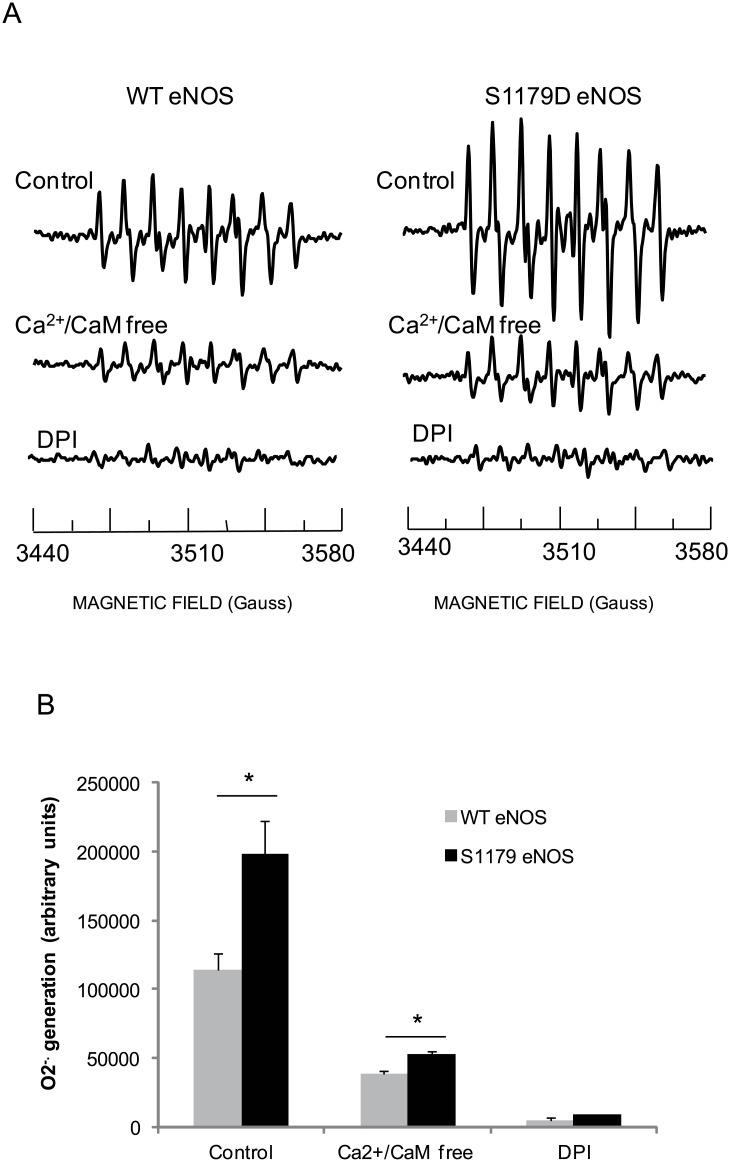
Ser1179D mutation enhanced both eNOS and reductase domain O_2_^.-^ generation capacity. 2μg eNOS protein mixed with 20 mM spin trap DEPMPO in 50 mM Tris-HCl buffer, pH 7.6 containing 0.5 mM NADPH in presence or absence of 0.5 mM Ca^2+^, 10 μg/ml CaM, the DEPMPO-OOH adducted were recorded by EPR at 3440 to 3580 Hzs. Data shown are means from three experiments. (A) Wild type eNOS produced a strong DEPMPO-OOH adduct signal in the Tris HCl buffer in the presence of 0.5 mM Ca^2+^, 10 μg/ml CaM (left up-trace). Compared to wild type eNOS, Ser1179D eNOS have significant higher DEPMPO-OOH adduct signal (Right up- trace). Depletion of Ca^2+^/CaM with 25mM EDTA significantly decreased O_2_^.-^ generation from WT eNOS (left middle trace) and from S1179D eNOS (right middle trace). Moreover, these DEPMPO-OOH adduct signals were completely inhibited by 10mM DPI, and FMN/FAD inhibitor (bottom traces); (B) Statistical analysis indicated that O_2_^.-^ generation from S1179D eNOS was significantly higher than that from WT eNOS (P<0.05, n = 5); Depletion of Ca^2+^/CaM significantly inhibited O_2_^.-^ generation capacity from both wild type eNOS and S1179D eNOS (n = 5, P<0.01).

Previous NO studies show that eNOS produces NO from a heme center in the oxygenase domain[32]. In addition to L-arginine, eNOS needs NADPH as an electron donor and many other co-factors such as Ca^2+^, CaM and BH4 to generating NO. ENOS generates O_2_^.-^ in the absence of L-arginine and BH_4._ In this case the electrons are delivered to heme center and oxygen is oxidized into O_2_^.-^. In this experiment we first examined whether Ca^2+^ and CaM affect O_2_^.-^ generation from WT eNOS and S1179D eNOS. EPR spectra showed that there was a strong DEPMPO-OOH adducts signal for WT eNOS. ENOS still generates a significant amount of O_2_^.-^ in the absence of Ca^2+^ and CaM (38971.5±2069.9, 34.2±1.8% compared to that from WT eNOS in presence of Ca^2+^ /CaM). In Ca^2+^ and CaM depleted conditions, S1179D eNOS also generated a significant amount of O_2_^.-^ (53186.5±1520.67, 26.8±0.7% comparing to S1179D eNOS control. (Fig 4A middle traces). These results indicate that there may be another functional center producing O_2_^.-^ outside of the heme center as the electron was not delivered to heme center. A potential O_2_^.-^ generation site outside oxygenase domain is the FMN and FAD/NADPH center, which locates in the reductase domain of eNOS[11]. As WT eNOS and S1179D eNOS were treated with 10mM DPI, a flavoprotein inhibitor, and the DEPMPO-OOH signals significantly decreased (Fig 4A bottom traces and 4B).

### L-Arginine, BH_4_ Did Not Affect the O_2_^.-^ Generation from eNOS Reductase Domain

**Fig 5:**
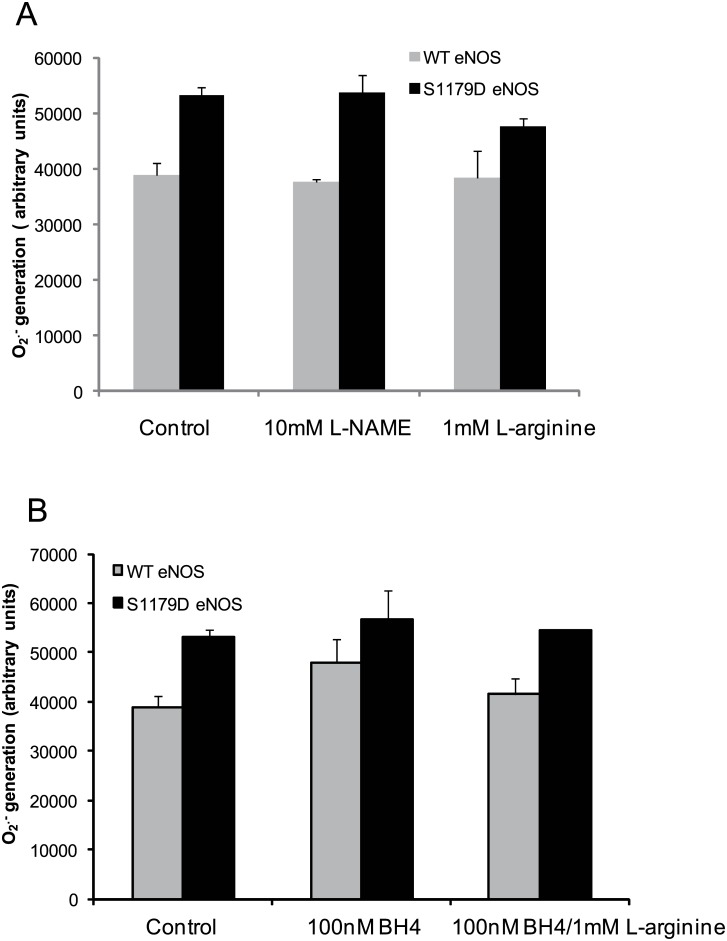
Both BH_4_ and L-arginine do not affect the O_2_^.-^ generation from eNOS in the absence of Ca^2+^/CaM. After 2μg eNOS protein mixed with 20 mM DEPMPO in 50 mM Tris-HCl buffer, pH 7.6 containing 0.5 mM NADPH and depletion of Ca^2+^ /CaM by adding 20mM EDTA, the DEPMPO-OOH adduct signal were recorded by EPR at 3440 to 3580 Hzs. (A) At depletion of Ca^2+^/CaM condition, the effect of L-arginine (1 mM) or L-NAME (10mM) on superoxide generation from eNOS. (B) At depletion of Ca^2+^ /CaM condition, the effect of BH4 (100 nM) or BH4 and L-arginine (1 mM) together on superoxide generation from eNOS. Data shown were the mean values from four independent experiments.

To exclude the potential effect of the oxygenase domain on the O_2_^.-^ release from reductase domain, we used 1mM L-NAME, a heme center inhibitor. L-NAME did not cause a significant inhibitory effect on O_2_^.-^ generation from WT eNOS or S1179D eNOS when compared to control. The DEPMPO-OOH adduct signals from WT eNOS and S1179D eNOS were 37661± 575.2 and 53783 ± 2958.5, respectively. When WT eNOS and S1179D eNOS were treated with L-arginine (1mM), a substrate for NO generation of eNOS which also binds in heme center, the DEPMPO-OOH adduct signals generated were 38367±4841.58 and 47520±1533.9 respectively, with no significant difference when compared to controls (Fig 5A).

Early studies showed that BH4 and L-arginine were the determinant factors regulating NO or O_2_^.-^ generation from eNOS heme center[32–33]. Next we investigated whether BH4 or BH4 and L-arginine together affected O_2_^.-^ release from the reductase domain. WT eNOS and S1179D eNOS were incubated BH4 (100nM) at a Ca^2+^/CaM depletion condition. As shown in Fig 5B, the DEPMPO-OOH signals generated from WT eNOS and S1179D were 47930.4±4679 and 56564.81±5964.4, respectively. When WT eNOS and S1179D eNOS were incubated with BH4/ L-arginine (100nM/1mM), the DEPMPO-OOH signals generated from WT eNOS and S1179D were 41459.65±3403.8 and 54607.8±50.7, respectively (Fig 5B). There was no significant difference between BH4 treated groups and BH4/ L-arginine groups in the absence of Ca^2+^ and CaM.

### The Affirmative Evidence of O_2_^.-^ Generation from the eNOS Reductase Domain

**Fig 6:**
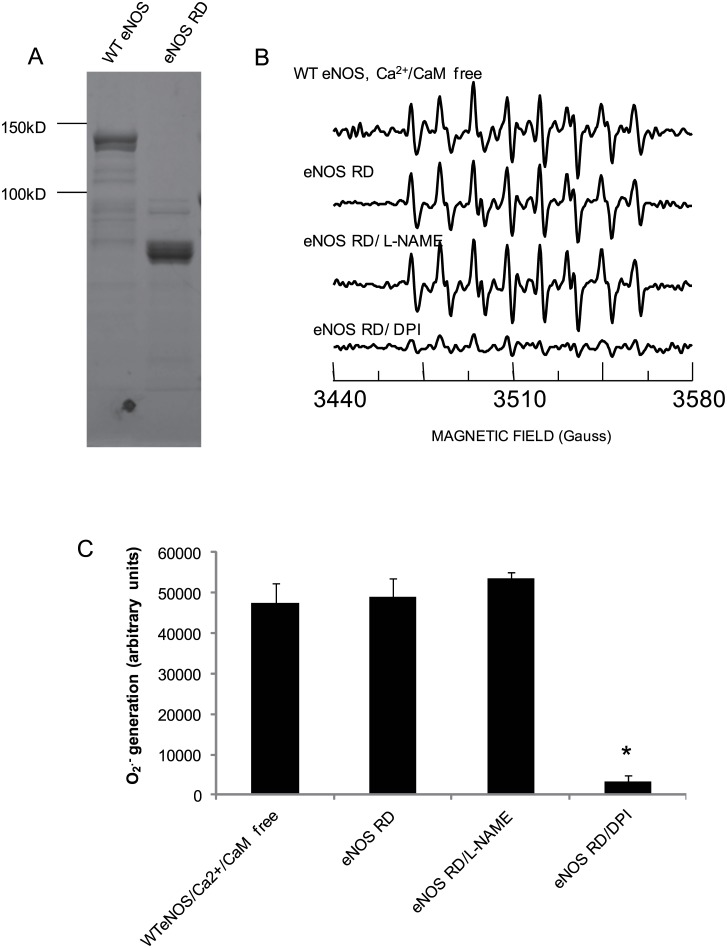
O_2_^-.^ generates from the cloned eNOS reductase domain. (A) The recombinant DNA of Bovine eNOS reductase domain was over-expressed in E.Coli as described in materials and methods. After purified, the purity was identified on SDS-PAGE gel by Coomassie brilliant blue staining. (B) EPR spectra showed that DEPMPO-OOH adduct signal generated from 2μg purified WT eNOS at condition of Ca^2+^/CaM depletion (*Top trace*); Equal amount of recombinant eNOS reductase domain protein produced equal strong DEPMPO-OOH adduct signal as WT eNOS in the absence of Ca and CaM (*Second trace*, *RD*); the signal did not inhibited by 10mM L-NAME but inhibited by 10mM DPI (*Third trace and bottom trace*). (C) Statistic analysis confirmed that there was not significant different of O_2_^-.^ generated from WT eNOS and from recombinant eNOS reductase domain protein and both were inhibited by 10mM DPI. Values are mean± S.E., n = 3–5 determinations.

To further determine the mechanism of O_2_^.-^ release from the eNOS reductase domain, we subcloned the bovine eNOS reductase domain from WT eNOS plasmid and expressed it in E.Coli. 1μg purified eNOS reductase domain protein was identified on the SDS-page gel (Fig 6A). Compared to WT eNOS without Ca^2+^ /CaM, 2μg purified eNOS reductase domain protein generated an equal amount of O_2_^.-^ (Fig 6B top trace and second trace, respectively). The DEPMPO-OOH adduct signals were 47392±5090.3 and 49124.05±4332.34, respectively. This O_2_^.-^ generation from eNOS reductase domain was significantly blocked by the FMN/FAD inhibitor, DPI; but not by the heme inhibitor L-NAME (Fig 6B fourth trace and third trace, and Fig 6C).

### NADPH-Consumption Evidence for O_2_^.-^ Generation from eNOS Reductase Domain and Regulation by Phosphorylation

**Fig 7:**
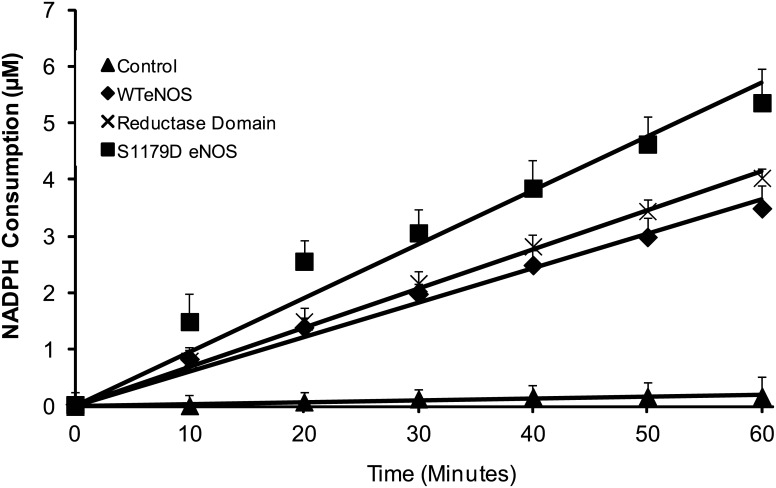
NADPH consumption by eNOS reductase domain. NADPH oxidation was monitored spectrophotometrically at 340 nm in the reactions containing 50 mM Tris-HCl, pH 7.4, 60 μM NADPH, 32 μg/ml eNOS. As shown, self- oxidation of NADPH was 0.3 μM per hour in the absence of Ca^2+^/ CaM (*triangle*). 2μg WT eNOS and purified recombinant eNOS reductase domain protein consumed 4.5uM NADPH per hour and 4.1 μM NADPH per hour in the absence of Ca^2+^/ CaM, respectively (*diamond and cross*). However, S1179D eNOS caused 6.3 μM NADPH oxidation per hour in the absence of Ca^2+^/ CaM (*rectangle*). Data shown are the means of the results from three independent experiments.

As eNOS needs to consume NADPH to generate free electron, NADPH consumption is the indirect index consisting with O_2_^.-^ generation rate. To further exclude other potential O_2_^.-^ generation centers in eNOS, we next compared the NADPH consumption of WT eNOS, S1179D eNOS and purified eNOS reductase domain. At condition of Ca^2+^ and CaM depletion, there was not significant difference of NADPH consumption rate between WT eNOS and eNOS reductase domain (3.49± 0.39μM/h and 4.03± 0.17 μM/h for WT eNOS and eNOS reductase domain respectively, P>0.05). However, S1179D eNOS consumed a significantly higher amount of NADPH than WT eNOS and eNOS reductase domain (5.35± 0.58 μM/h, P<0.05 compare to WT eNOS and reductase domain, respectively. Fig 7).

## Discussion

In this study, we obtained three important findings. First, spin trapping experiments presented definitive evidence that eNOS generates O_2_^.-^ from the FMN and FAD/NADPH binding site of the reductase domain. Second, O_2_^.-^generation from eNOS reductase domain is not regulated by eNOS cofactors BH4, L-arginine, and Ca^2+^ /CaM. However, O_2_^.-^ generation from eNOS reductase domain is regulated by eNOS phosphorylation. Third, purified eNOS reductase domain protein provided direct evidence that the O_2_^.-^ generation center is located in the FMN and FAD/NADPH binding sites of the eNOS reductase domain. Finally, NADPH consumption rate also indicate that eNOS reductase domain has an electron leakage site and generation of O_2_^.-^.

Although eNOS, nNOS and iNOS have different oxygenase domains and enzyme activities, they have similar structure in their reductase domains those function like NADPH-cytochrome P-450 [13]. Previous studies showed that electron flux leaked from iNOS reductase domain and was not affected by L-arginine[12]. Also, nNOS HSP90 binding studies showed that HSP90 could largely but not completely inhibit O_2_^.-^ generation from nNOS[34–35]. Pharmaceutical inhibitors study showed that nNOS reductase domain may also involve in O_2_^.-^ generation from nNOS [36]. Moreover, a recent study showed that S-glutathionylation of Cysteine 689 and Cysteine 908 in human eNOS increased O_2_^.-^ generation from eNOS. This O_2_^.-^ generation was speculated from eNOS reductase domain as they locate in the FAD/NADPH binding subdomain[11]. In the current study, combining DEPMPO spin trapping with purified eNOS reductase domain protein, we have for the first time offered direct evidence that O_2_^.-^ generated from the FMN and FAD/NADPH binding sites of eNOS. Overall, previous studies and our current study indicated all three isoforms of NOSs generate superoxide from their reductase domain.

As eNOS function is controlled by many factors, including BH4, L-arginine and Ca^2+^ /CaM, we also investigated the impact of these factors on the function of the eNOS reductase domain. Distinct from O_2_^.-^ generation from the heme center of the eNOS oxygenase domain, BH4, L-arginine and Ca^2+^ /CaM did not affect O_2_^.-^ generation from eNOS reductase domain. As DPI, a flavoprotein inhibitor, inhibited O_2_^.-^ generation, the generation site may be the flavin center of the FMN and FAD/NADPH binding subdomains.

Unlike the effect of eNOS cofactors on eNOS reductase domain, eNOS Ser1179 phosphorylation enhances O_2_^.-^ generation from eNOS reductase domain. Our results show that mutation of Ser1179D eNOS significantly increases O_2_^.-^ generation from both eNOS oxygenase domain and eNOS reductase domain (Fig 4). NADPH consumption rates also confirm that eNOS Ser1179 phosphorylation enhances electron generation and electron delivery efficiency, which is indicative of more oxygen molecules converting into O_2_^.-^. We are on the way to determining the importance of electron redistribution between the eNOS oxygenase domain and eNOS reductase domain caused by eNOS Ser1179 phosphorylation.

**Fig 8:**
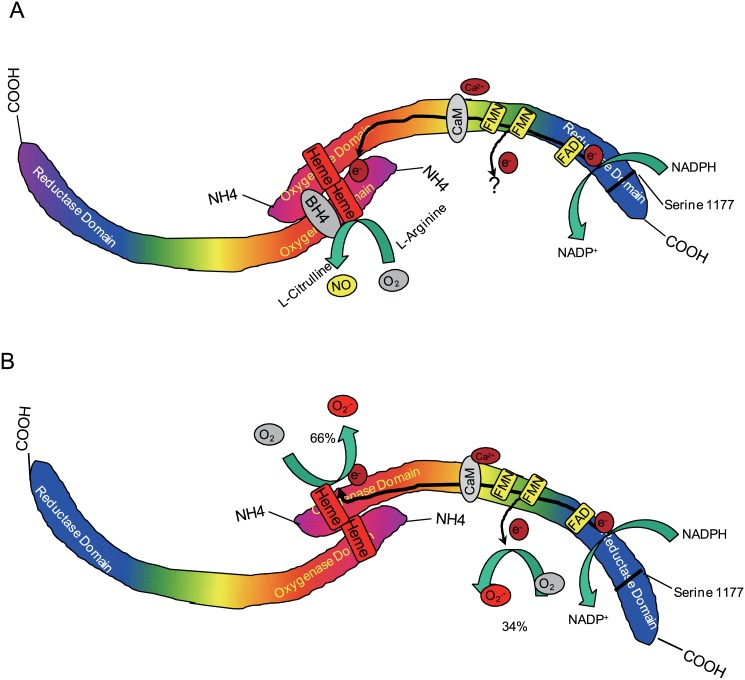
Schematic diagram illustrating the O_2_^.-^ generation mechanisms from eNOS reductase domain and oxygenase domain. In addition of heme center of eNOS oxygenase domain (N-terminal), O_2_^.-^ also generates from eNOS reductase domain. This O_2_^.-^ generation center locates in the FMN and FAD/NADPH binding site. (A) At BH4 and L-arginine binding condition, electron generated from eNOS reductase domain is delivered into heme center of another eNOS oxygenase domain and generates NO. By binding with FMN or FAD, electron leakage in FMN and FAD/NADPH binding site is also inhibited; (B) However, at uncouple condition (BH4 and L-arginine depletion), the electron generated from eNOS reductase domain is delivered into same eNOS oxygenase domain and generates O_2_^.-^. Moreover, the electron leakage at FMN and FAD/NADPH binding sites also results in O_2_^.-^ generation. The O_2_^.-^ generation capacity in heme center of oxygenase domain and flavin center of reductase domain are 65.8% and 34.2% respectively.

Previous observations have found that the eNOS reductase domain binds with NADPH and generates an electron, which is delivered into the heme center of the oxygenase domain[32]. In this study, our results clearly show that FMN and FAD/NADPH binding sites of the eNOS reductase domain also generates O_2_^.-^. At physiological conditions, O_2_^.-^ generation from the oxygenase domain and reductase domain in WT eNOS are 65.7 ± 10.3% and 34.2±1.8%, respectively (Figs 2 and 4). Similar NADPH consumption rate by WT eNOS in absence of Ca^2+^/CaM and by purified eNOS reductase domain also indicated that the electron was consumed by eNOS reductase domain (Fig 7). Although we did not determine the exact electron leakage site in FMN or FAD/NADPH binding subdomains, our DPI inhibitor results clearly indicate the importance of FMN or FAD/NADPH binding sites for eNOS functions. Considering a significant amount of O_2_^.-^is generated in the eNOS reductase domain (1/3 of total O_2_^.-^generation from eNOS, Fig 8), it may play critical role in maintenance of eNOS function and dimer structure. While we have identified this new O_2_^.-^generation center in the eNOS reductase domain, its potential binding cofactor or chaperone protein remain unclear.

## Conclusion

In conclusion, we offer direct evidence to identify a new O_2_^.-^ generation site in the eNOS reductase domain. This site is not regulated by BH4, L-arginine and Ca^2+^/CaM, but it is enhanced by eNOS phosphorylation. The O_2_^.-^ generation capacity of the eNOS reductase domain is around 1/3 of the total eNOS O_2_^.-^ generation capacity. Studies with pharmacologic inhibitors and cloned eNOS constructs indicate that this O_2_^.-^ generation site is located in the FMN and FAD/NADPH binding region of eNOS reductase domain. These novel findings will aid the understanding of eNOS function and may help in the development of new therapeutic strategy for the patients suffering eNOS dysfunction related diseases.

## Abbreviations

BH_4_: Tetrahydrobiopterin
CaM: Calmodulin
DPI: Diphenyleneiodonium
DEPMPO: 5-diethoxyphosphoryl-5-methyl-1-pyrroline-*N*-oxide
eNOS: Endothelial nitric oxide synthase
eNOS RD: eNOS reductase domain
EPR: Electron paramagnetic resonance spectroscopy
FPLC: Fast Performance Liquid Chromatography
O_2_^.-^: Superoxide
WT eNOS: Wild type eNOS
S1179D eNOS: Serine1179 aspartate eNOS mutant
NADPH: Nicotinamide adenine dinucleotide phosphate
